# PIECES of My RELATIONSHIPS: The Cultural Adaptation of a Biographical Assessment Tool for Indigenous Older Adults in Canada

**DOI:** 10.1093/geront/gnad176

**Published:** 2023-12-27

**Authors:** Kristen Jacklin, Karen Pitawanakwat, Melissa Blind, Dana Ketcher, Louise Jones, Emily Piraino, Monica Bretzlaff

**Affiliations:** Memory Keepers Medical Discovery Team, Department of Family Medicine and Biobehavioral Health, University of Minnesota Medical School, Duluth, Minnesota, USA; Naandwechige-Gamig Wikwemikong Health Centre, Wikwemikong, Ontario, Canada; Memory Keepers Medical Discovery Team, Department of Family Medicine and Biobehavioral Health, University of Minnesota Medical School, Duluth, Minnesota, USA; Memory Keepers Medical Discovery Team, Department of Family Medicine and Biobehavioral Health, University of Minnesota Medical School, Duluth, Minnesota, USA; Independent Researcher, Halifax, Nova Scotia, Canada; Psychogeriatric Resource Consultant, Algoma, NEBSO Indigenous Engagement Strategy lead, North Bay Regional Health Centre, North Bay, Ontario, Canada; Behavioural Supports Ontario (Provincial & North East), Seniors’ Mental Health Integrated Service, Seniors’ Mental Health-Regional Consultation Service, North Bay Regional Health Centre, North Bay, Ontario, Canada

**Keywords:** Culturally appropriate assessments, Cultural competence practice, Cultural safety, Indigenous populations, Institutional care/residential care

## Abstract

**Background and Objectives:**

Healthcare services are rarely designed to meet the needs of Indigenous people, resulting in culturally unsafe care and assessment tools. This paper describes a collaboration between North East Behavioural Supports Ontario (NEBSO), university researchers, and Indigenous communities to adapt a biographical assessment tool used by NEBSO to be culturally appropriate and safe for Indigenous older adults (55+) in long-term care facilities in Ontario, Canada.

**Research Design and Methods:**

Over 36 months, this project applied an Indigenized, community-based participatory research (CBPR) and cultural safety framework to the adaptation process. Qualitative data sources include the guidance of an Indigenous Elder, an Anishinaabe Language Expert Group, and focus groups conducted along the North Shore of Lake Huron, Sudbury, and Cochrane, Ontario.

**Results:**

The adapted tool shifts the focus from personhood to relationships, includes culturally relevant domains, and supports trauma-informed approaches. Five themes were identified during the adaptation process: (1) practicing a relational approach to care, (2) valuing Indigenous language, (3) understanding Indigenous trauma, (4) respecting cultural values and understandings, and (5) addressing systemic barriers to culturally safe care.

**Discussion and Implications:**

Themes elucidated from this research process can inform future studies adapting mainstream practice tools and developing new tools for Indigenous populations. The collaboration and approach to this adaptation process demonstrated how cultural safety at systemic and practice levels can be addressed through CPBR partnerships between universities, organizations, and Indigenous communities. Findings support the need to evaluate the cultural safety of other assessments for older Indigenous adults in health care settings.

## Background and Objectives

As the incidence and prevalence of dementia in Indigenous populations have risen and surpassed those of the general population (Jacklin et al., 2013; Moon et al., 2023), behavioral health interventions to support such diagnoses are increasingly needed. The availability of culturally appropriate institutional care for Indigenous older adults is a longstanding challenge for Indigenous families in North America (Jervis, 2010). Although health disparities ultimately must be addressed at multiple levels, creating culturally safe and appropriate health services is crucial to improving health outcomes (Greenwood & de Leeuw, 2012). The research described here is concerned with making health care interactions culturally safe for aging and older Indigenous individuals (55+) and their families. Specifically, we focus on older adults with, or at risk of, responsive behaviors/personal expressions associated with dementia, complex mental health challenges, substance use, and/or other neurological conditions.

Culturally safe care and tools are considered best practice solutions for situations in which colonialism affects clinical experience (Hole et al., 2015). It is an approach that can address intergenerational trauma and, ultimately, social determinants of health inequity in Indigenous populations (Kim, 2019). The concept of cultural safety was originally introduced in Aotearoa New Zealand by Māori nurses with a focus on structural inequities and power imbalances arising from the colonial relationship (Papps & Ramsden, 1996) and has been useful in addressing health care inequities in other countries such as Canada (Baker & Giles, 2012; Jacklin et al., 2020; Smye & Browne, 2002; Webkamigad, Cote-Meek, et al., 2020). Cultural safety is a distinct approach that explicitly targets power differentials and organizational practices through a critical self-reflective lens (Curtis et al., 2019) and asks health care providers to take responsibility for recognizing vulnerable service users and introducing culturally safe care into their practices (Baker & Giles, 2012).

Older Indigenous adults and their families often report culturally inappropriate and unsafe experiences with health care services offered to the general population (Allen & Smylie, 2015; Griffin-Pierce et al., 2008; Jacklin et al., 2015). The ongoing need for culturally safe care coupled with the unique service needs of older Indigenous adults presents an opportunity to develop and adapt specialized tools, services, and care strategies in partnership with this population. Our aim is to adapt a biographical assessment tool (PIECES of my PERSONHOOD) used by direct behavioral health care services with Indigenous populations in northeastern Ontario using an Indigenized community-based participatory research methodology (CBPR) approach to determine what elements tool could be changed to improve the cultural safety of the tool for Indigenous populations.

## Research Design and Methods

This project was primarily grounded in a combination of Indigenous methodologies and the pillars of CBPR. This approach is based on the team’s previous work adapting and creating culturally safe materials (Jacklin et al., 2020; Webkamigad, Cote-Meek, et al., 2020). This included incorporating Indigenous ways of knowing, privileging stories, and storytelling as a culturally informed interpretation process, situating oneself in the research, taking the time to build relationships and follow Indigenous protocols, and being accountable to the communities we work with by adhering to the 4 R’s of research: Respect, Relevance, Reciprocity, and Responsibility (Absolon, 2010; Kirkness & Barnhardt, 1991; Kovach, 2021; Wilson, 2008). The CBPR framework facilitated partnership building between North East Behavioural Supports Ontario (NEBSO) and Indigenous health organizations; relationship development between NEBSO staff, university researchers, and community members; and the incorporation of an Anishinaabe Research Advisory Council, Elder Advisor, and an Anishinaabe Language Expert Group (ALEG). We relied on reputational case selection for these advisory roles to ensure the appropriate selection of participants (LeCompte & Schensul, 2010).

Elder Advisor Jerry Otowadjiwan (Wiikwemkoong Unceded Territory) participated as a co-researcher and provided spiritual guidance, cultural knowledge to inform the tool development, Indigenous teachings, language translation, and advice. The Anishinaabe Research Advisory Council includes Anishinaabe Elders, caregivers, and health care providers from the seven First Nations on Manitoulin Island who had been previously established to support ongoing dementia research with Indigenous populations (Jacklin et al., 2020). The ALEG included nine members (four male, five female) who are fluent Anishinaabemwin language speakers from the seven First Nations on Manitoulin Island, Ontario. They are known in their communities for their knowledge of the language and deep understanding of culture. Centering these Indigenous Elders throughout this process was essential to this project and works to decolonize and Indigenize the research process and outcomes (Crouch (Deg Hit’an, Coahuiltecan) et al., 2023).

The original tool to be adapted, PIECES of my PERSONHOOD, was developed by NEBSO in 2012 via a quality improvement Kaizen approach (Health Quality Ontario, 2013) and is used across Ontario in long-term care settings to collect information on an individual’s life experiences, important relationships, personal preferences, and other psychosocial and environmental factors that influence their daily lives (North Bay Regional Health Centre, 2012). The PIECES of my PERSONHOOD tool includes 21 sections of biographical domains to enhance person-centered care (Table 1). Personal biography is one of several aspects of care prioritized by NEBSO teams when a referral is received, and it fits into the larger holistic PIECES framework (Hamilton et al., 2010) that guides all NEBSO assessments and seeks to identify unmet **P**hysical, **I**ntellectual, and **E**motional needs as they interact with the person’s **C**apabilities, **E**nvironment, and **S**ocial history.

**Table 1.** Adaptation and Restructuring of Domains in PIECES of My PERSONHOOD Tool

Original: PIECES of My PERSONHOOD	Adaptation: PIECES of my RELATIONSHIPS	Adaptation: Family Supplement
Preferred name	**Who I Am** *Greeting, my name is … what is yours?* *Is this the name you use most often?* *Where did you come from, can you tell me?* *Where were you born?* *Who are your parents?* *Have you ever raised or taken care of an animal? What did you call them? What was their name?* *Who is the one who looks after all your affairs?* *Is there anyone who comes around to take you on outings?* *Who is it that tells you not to climb on stairs to reach up high on your own?*	*Was your loved one always loved?* *Were they ever treated poorly by a partner?*
Preferred language		
Family background		
Pets/names		
Significant persons in life/ relationship		
Mealtime preferences	**A Day in My Life** *Are you happy to eat with others?* *What foods taste good to you/don’t taste good to you?* *Are there any foods that don’t look or feel good in your mouth?* *Do you sleep well?* *When do you go to sleep, or lay down?* *What do you do to prepare before going to sleep?* *When do you wake up, or get up?* *What do you like to do when you get up in the morning?* *Are you happy with visitors?* *What do you enjoy doing while visiting? (Watching TV, playing cards, something else?)* *What do you try doing when you are feeling lonely?*	
Sleep/wake preferences		
Socialization preferences		
Personal preferences		
I am most proud to be known as/for…	**What Keeps Me Going** *Of all the things that kept you busy in your life, what did you enjoy the most?* *How about in the spring? Summer? Fall? Winter?* *What are you still doing now?* *What kinds of things were you very good or gifted at?*	*What kinds of things are they very good or gifted at?* *How are they able to still give back?*
Life role/previous occupation		
Interests/hobbies		
Significant high point(s) in life		
Significant dates and meaning		
Sources of: hope/comfort/joy/ inspiration/favorite things		
Significant low point(s) in life/ trauma	**How to Keep Me Safe** *What memories do you carry in your heart?* *Is there something that you have a fear of that scares you? (Like a spider, snake, or other superficial fear?)* *Do you have a deep-down fear? (Like the sound of thunder?)* *Do you ever use smudging to clean your home?* *Are there sounds you don’t like?* *Is there anything you find bothers you, makes you uneasy, worried, or frustrated?* *How can others tell when you’re feeling sad, frustrated, worried, or lonely?*	*Is there anything that bothers your loved one, makes them uneasy, worried, or frustrated?* *How can others tell when your loved one is feeling sad? Frustrated? Angry? Worried? Lonely?* *What are the things that can be done or said to help your loved one feel comfortable?* *Does your loved one ever have to use smudging to clean their home?*
Coping mechanisms/validation phrases		
Dislikes/fears		
Significant dates and meanings		
Expression of emotions		
Spirituality, religion, and traditions	**How I Care for my Whole Being** *What are some of the things you did when you were sick?* *What about now?* *What are some of the ways you express you are thankful?* *Do you need some sort of support when you pray? (With rosary, tobacco, traditional offering, syllabic hymn books, gospel prayers, signing songs in your language, prayers in your language?)* *When do you prefer to be alone?*	*Does your loved one need some sort of support when they pray? (With rosary, tobacco, traditional offering, syllabic hymn books, gospel prayers, signing songs in your language, prayers in your language?)*

*Notes:* To the left are the domains from the original PIECES of my PERSONHOOD tool. In the middle column, new domains have been created to combine original domains. Questions are included in the new tool to assist workers in guiding the conversation. The final column includes questions that loved ones may be uncomfortable answering due to the personal nature of them or because of the cultural value of humility.

**Figure 1.**
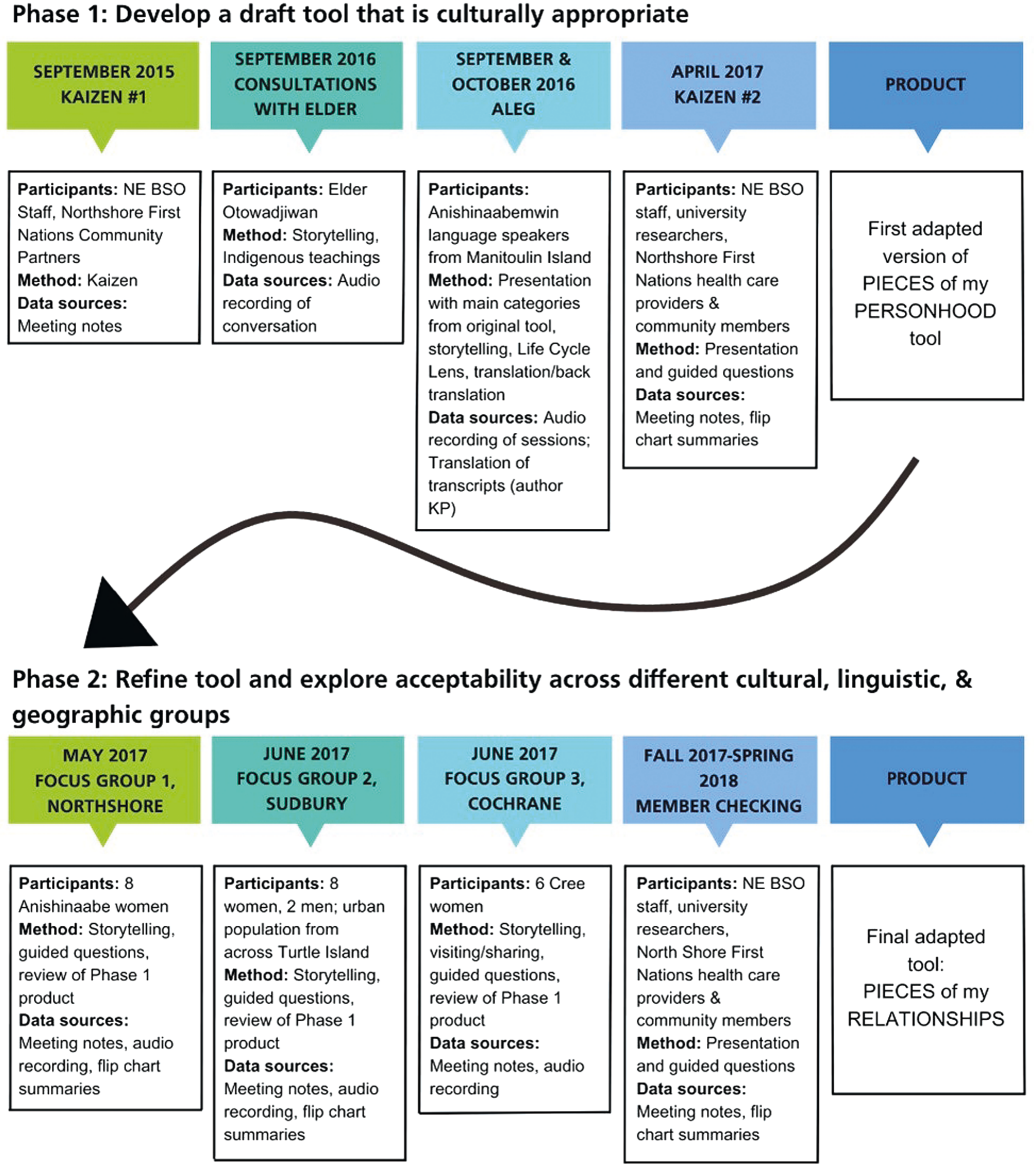
PIECES of my PERSONHOOD adaptation process.

This project was divided into two phases. The objective of Phase 1 was to gain a better understanding of the concepts discussed in the original tool and develop a draft adapted tool that would be more culturally appropriate. Phase 1 included discussions with the Elder Advisor, a Kaizen session, and meetings with the ALEG. The research team included both Indigenous and non-Indigenous members. Co-researchers included the Elder Advisor, the Kaizen working group, and the ALEG. Kaizens are part of the NEBSO culture and have been used by them in the past as a part of the Lean health care methodologies related to quality improvement (Jackson, 2017). It uses a structured approach used to a specific problem and creates a plan for action and engagement. For NEBSO, the problem was that Indigenous communities were not utilizing their services. The objective of Phase 2 was to further refine the tool and explore acceptability across different cultural and linguistic groups in rural, urban, and remote areas of Ontario with research participants in focus groups (Figure 1). The Anishinaabe Research Advisory Council met monthly in both phases and provided overall guidance to the process.

### Data Collection

**Figure 2.**
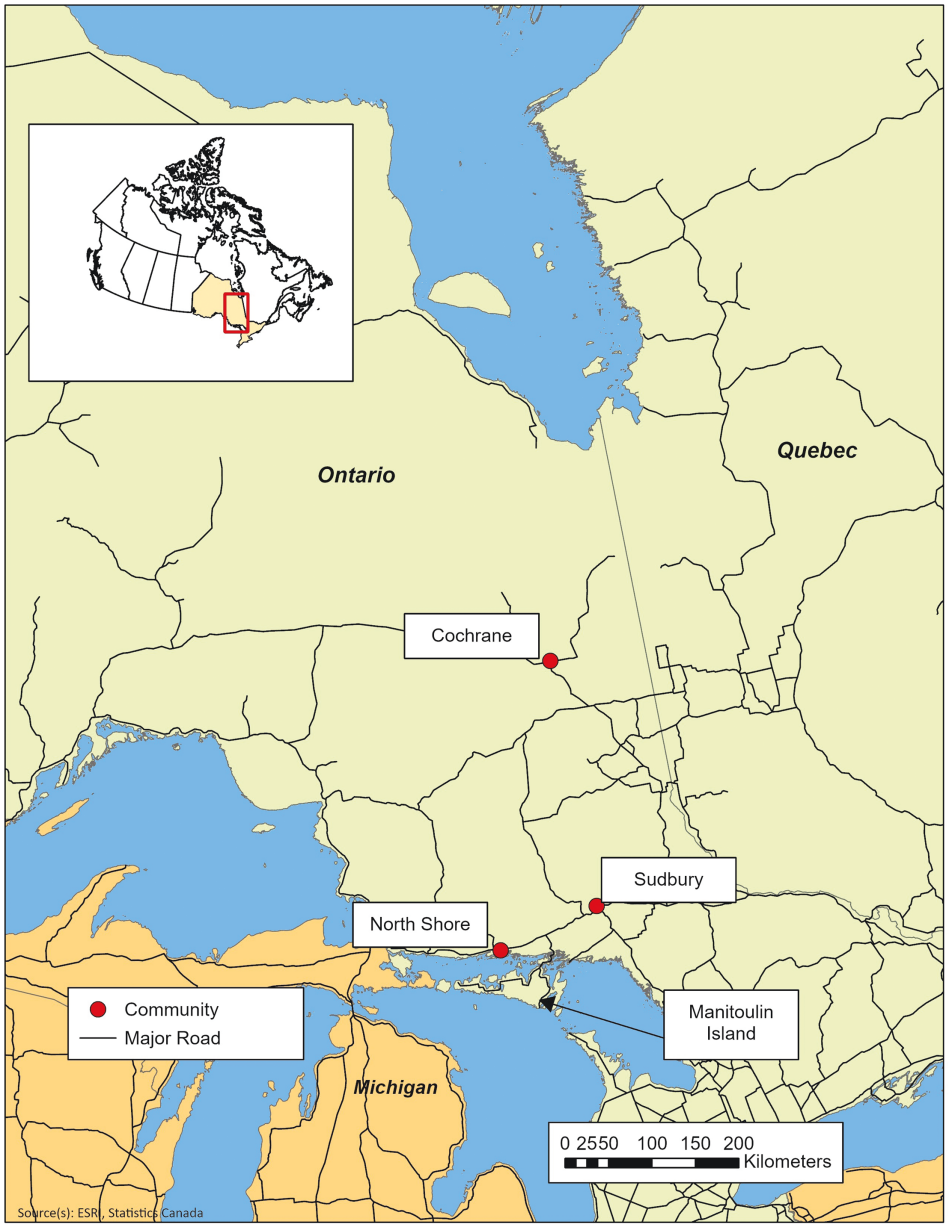
Focus group locations in northeastern Ontario.

Data collection was sequential (see Figure 1) and designed to support an adaptation of the PIECES tool that would be relevant to diverse Indigenous peoples living in northeastern Ontario (primarily Cree, Oji-Cree, and Ojibway) (Figure 2). Phase 1 began with a Kaizen involving NEBSO staff, researchers, health care providers, and community members. At the Kaizen, suggestions concerning the wording of the questions were considered and Indigenous knowledge was prioritized in decision making. We agreed upon an integrated Indigenous and Western approach to the research framework and plans were established to form a working group, consisting of NEBSO staff and the research team, to fully adapt the tool to be culturally safe and appropriate.

Next, the original PIECES of my PERSONHOOD tool was used to facilitate discussions with the Elder Advisor and the ALEG, which centered on appropriateness of the questions, acceptability of the wording, and potential translation issues. The Elder Advisor provided initial guidance on how to approach an Elder and start the process of asking questions. Conversations with the ALEG were guided by a PowerPoint presentation that had each of the sections of the original tool and the corresponding questions from that section. We asked the following questions: Does this question make sense in your area? How would you ask this? Would you be comfortable answering this? Why or why not? How would you talk about [category] with older people? Using storytelling the ALEG shared stories related to the topic areas, described how Elders might respond if asked these questions, and provided specific guidance on rewording the questions.

A second Kaizen was held in April 2017 to build consensus on a revised draft tool based on data from the original Kaizen, the Elder Advisor, and ALEG consultations. During this second Kaizen, the working group gave a PowerPoint presentation on the adaptation process with the Elder Advisor and ALEG. The Kaizen participants then went through the tool question by question with discussions centered on storytelling to help the group decide on the wording that would be used for the focus groups. The revised version of the tool was used to facilitate data collection during Phase 2.

In Phase 2, we conducted focus groups in three regions in northeastern Ontario to represent linguistic, geographic, and cultural diversity: the North Shore of Lake Huron (Anishinaabe), Sudbury (Anishinaabe and diverse urban), and Cochrane (Cree; Figure 2). Focus group participants were recruited from the community or region served by the partner community organization with the assistance of local community staff. Inclusion criteria required participants to be a minimum of 18 years old and self-identify as an Indigenous older adult (self-defined), caregiver, or health care provider working with Indigenous older adults. A total of 24 participants joined the groups: 8 women from the North Shore (4 Indigenous language speakers), 10 participants from Sudbury (8 women, 2 men), and 6 women from Cochrane (4 Indigenous language speakers). Consent was obtained from each participant prior to participation in the focus groups using an information package presented by the researcher and a written consent form.

Focus groups incorporated Indigenous approaches to research. Traditional Knowledge Keepers in each region were presented with a tobacco tie and an honorarium to open and close the focus groups with a prayer and help guide the research “in a good way” (Flicker et al., 2015, p. 1149). Tobacco is one of the four sacred medicines and is used as an offering to ask for guidance. A tobacco tie is one method of offering tobacco and uses a piece of cloth that holds the tobacco to create a small sacred bundle that can be offered to the Traditional Knowledge Keeper.

After the opening prayer, the research team introduced themselves and their role in the research. Identifying ourselves and our roles in relation to the Spirit is congruent with establishing respectful practice within an Indigenous worldview (Absolon, 2010). Participants were invited to introduce themselves and share as much as they felt comfortable with. The research team and participants shared a meal and participants were given an honorarium of $50 CDN and reimbursed for travel expenses if needed. This is consistent with Indigenous methodologies and creating a safe environment where Indigenous people can share their experiences and stories (Kovach, 2021; Wilson, 2008).

Focus groups were facilitated by authors KP, LJ, and EP. Facilitators handed out copies of the revised tool and read each question aloud and asked for feedback from the group. Facilitation questions were the same questions used during the second Kaizen meeting during Phase 1.

All focus groups were audio recorded. A member of the research team (LJ) transcribed the audio. In cases where Anishinaabemwin was used, author KP, a fluent language speaker, and Anishinaabe Kwe from Wiikwemkoong Unceded Territory, provided the translation and transcription.

### Data Analysis

Analysis and the identification of themes was an inductive and iterative feedback process involving all research partners. During focus groups, participant comments and recommendations were recorded by the research team on large sticky notes for all participants to see as a form of *in the moment* member checking and validation (Charmaz, 2014; Lincoln & Guba, 1985). After each session with all participant groups, the research team members debriefed to analyze the information shared. This involved identifying key themes from each session and a discussion of how this information would inform the adaptation of the tool. Themes were used to create real-time consensus-based revisions to the tool which were then built upon in an iterative process at the next meeting or focus group, resulting in an Indigenized consensus model that draws on Glaser’s “state-of-the-art” method (1980). As a final step, the Anishinaabe Research Advisory Council and ALEG reviewed and confirmed the findings, sometimes offering additional information on the findings. Member checking and validation were completed by sharing the results back to focus group participants and interested community members. The resulting themes presented here were consolidated from the iterative process by the authors and validated by the Anishinaabe Research Advisory Council as a final step. The themes represent the cumulative and negotiated knowledge from each of the data collection points. This data analysis process seeks to place participants on equal footing as researchers, as “agentive” and “responsible theorists of their own experience” (Harvey, 2015, p. 34), aligning with CBPR and Indigenous methodological approaches.

### Ethics

Ethical approval and oversight were provided by the Laurentian University Research Ethics Board (#6009500) in Sudbury, Ontario, and by the Manitoulin Anishinaabek Research Review Committee. There were no community-based research ethics committees in the other participating regions.

## Results

The adaptation process took 36 months. We identified five consensus themes that were central to creating a culturally safe adaptation of the PIECES of my PERSONHOOD tool: (1) practice a relational approach to care, (2) value Indigenous language, (3) understand Indigenous trauma, (4) respect cultural values and understandings, and (5) address systemic barriers to culturally safe care (Supplementary Table 1). Each of these themes is explored in relation to the tool in the remainder of the results.

### Practice a Relational Approach to Care

An Indigenous relational worldview was central to the conversations among all participant groups. This worldview filtered into the discussions concerning the PIECES of my PERSONHOOD tool in distinct yet interconnected pathways: relationships with others and building therapeutic relationships.

#### Relationships with others

Participants highlighted three ways that the conceptualization and enactment of relationships can be specific to Indigenous worldview. The first is the direct relationships an individual has with other entities, including people, animals, land/environment, and spirits. The second is a person’s place in relation to the whole of those other entities. The third is the sacredness of relationships.

There was agreement across participant groups that in many Indigenous cultures, individuals are viewed in relation to others as opposed to more individualistic and Western views. The person being cared for may therefore give priority to identifying as one part of a greater whole rather than as an individual or person. This “whole” is expansive and could involve family, social networks, community, the land, and animals. This relational conception of the self was viewed to be in contrast with the notion of personhood. Similarly, the idea of one designated “caregiver” was viewed as a Western concept and instead, it was suggested that people receive support from several sources and individuals as the need arises. Elder Otowadjiwan stated, “It’s about belonging to a community.”

The importance of a relationship to animals was shared by an ALEG member who described a caregiving relationship between her family and a bear (Table 2). Focus group participants also shared that pets are considered special members of the family, sometimes referring to them as “little brothers” or “little sisters.” In every focus group, participants expressed joy at discussing animals, and suggested this as a lighthearted topic that could be used to get to know a loved one or to de-escalate a painful conversation.

**Table 2.** Illustrative Quotes of Themes During Adaptation Process Sessions

Theme	Sub theme	Supporting quotes
Practice a Relational Approach to Care	*Relationships with Others*	*My dad got a small bear who lost the parents. My dad was a trapper he found a small bear they brought it home, I saw the small box but he didn’t tell us what was in it. When my siblings and I went out we could hear the bear I thought it was a cat, but it was the bear. It was so small, we kept care of it and it got this big. The bear got so big that he could bat around his owner, the bear wanted to wrestle wanted to play. My dad used to take him to the small store everyone enjoyed feeding him ice cream and the bear really enjoyed this and pop. The pop used to come in bottles. He would drink it all and be given another one. Oh that’s enough he would be cautioned, no the bear didn’t listen. He really enjoyed little children coming around. The bear was never allowed to play though—he was kept tied up and taken around this way. One time we were eating over there, the little bear was outdoors tied up; there was a sound from outside, my dad wondered and went to investigate. Well the little bear had climbed up the veranda outside the house and fell off. All the boys ran outside and went to get the bear but there were no injuries. This happened twice the bear climbed up there. And he ate all kinds of food. When winter came my father went to the top of the hill where the pigs were kept there was an old hut he filled with earth and hay, this is where the bear was. The little bear looked like it was almost falling asleep that’s when they hibernate in the fall. This is where he was taken for all of winter, this is where he was. And when spring would come, we would be constantly running over back and forth, wondering when the little bear would come out. The bear got bigger and bigger ate more and more, my dad used to have to wear protective gear because the little bear wanted to wrestle. He wore gloves to hold him.* (Translated from Anishinaabemwin—ALEG Member)
	*Building Therapeutic Relationships*	*When you are going to meet somebody from a different area, I would properly introduce myself. Before I even ask, I tell them who I am* *…* *First of all, I give them my spirit name and my clan, and then I say, they call me by this name every day.* (Elder Otowadjiwan) *As soon as they nod their head, that means you have to be quiet for a little while, because they’re in thought. They want to put it in a way that is going to come out right, whatever they’re going to say. They’ll look down and stop for a while, then when they’re done, they’ll look at you.* (Elder Otowadjiwan) *When they start to speak, the elderly people, we have to stop. Just listen. No questions untilthey’re done. You’ll know when they’re done. They won’t say, “Okay, I’m done.” They won’tsay that. They’ll look down and stop for a while, then when they’re done, they’ll look at you.They won’t say nothing. They’ll just look at you. That means they’re finished.* (Elder Otowadjiwan) *We take those very highly, what we do in ceremony, and as to everyday, that follows you if you believe in what you follow. So you carry that with you. I carry that all the time. I don’t just leave it at ceremony. It has to come with me. And when we go and visit the elderly people, they have that too, but it’s [the visit] in a solid form.* (Elder Otowadjiwan)
Value Indigenous Language	*Cultural Differences in Word Meaning*	*In one of the language group meetings, I gave an example of how some older couples call each other old lady, old man, and one of the language speakers, a man, he said, you know some people will think that that’s rude and it really isn’t. It’s actually an honour to be called that. He said, do you realize the real translation of Mdimowenh? Mdimowenh is the old lady. And he goes, it’s really Nmindomowenh’im, your wife, this is the woman who carries my children, that’s what you’re saying in the language … So you lose those meanings for a while, and then somebody from another culture tells you that’s rude! I wouldn’t want to be known as an old man or an old lady. But that’s not the origin.* (ALEG Member)
Understand Indigenous Trauma	*Approach*	*You don’t want to bring that up if their spouse has been gone for ten years. You don’t want to bring that sadness up. If they want to bring it up, okay, but you let them bring it up. You let them lead.* (Sudbury Focus Group)
	*Triggers*	*Our Dad did not want to speak the language in the nursing home because of him being in residential school. There was a situation with one person that was not pleasant, and he did not want to speak the language. And he was very fluent.* (Sudbury Focus Group) *… so when I see this, “Where you were raised …” if you’re looking for the specific place you were raised … I was raised in residential school, away from my parents. I did my own [calculation]. How many years I was away from my parents and how many years I was actually raised by my parents. I went into residential school at the age of six. I became a mother at the age of 19. I was in residential school for seven years for ten months out of a year. Summertime was the only time I saw my parents. Sometimes Christmas if we were allowed to go … anyways, I calculated how many months, how many years I was actually raised by my parents from age six to age 19. When I started high school I had to leave. There was no high school … so I included those, from which I became I mother at 19. I had my first child at 19. When I calculated how many years I was actually with my parents, it was only 39 months. From age six to age 19 … I only actually lived with my parents 39 months.* (Cochrane Focus Group)
Respect Cultural Values and Understandings	*Cultural and Spiritual Knowledge*	*Some Aboriginal people like a smudge. It might help them if they are used to having a smudge every morning, or in the evening, or whenever. The first thing I do every morning when I come into work is I lay a smudge downstairs and the staff smudge there … that’s what I do. So if I did have that Alzheimer’s, that would help. I might not remember to do it, but as soon as I started I would know the feeling.* (Sudbury Focus Group) *The way we understand as Anishinabek the old ones and those who are preparing to leave for the spirit world, when this is not understood it gets very difficult to prepare. The one that is leaving is actually being stopped held back from continuing to walk their life the way it was meant to be. There are some who fear leaving and they need to be visited about how happy it is where they are going. They need to get to the place where they give up their life and believe they are ready. Sometimes they show this by refusing medication. Uncle Ben’s niece told the nursing home staff he was preparing to go, and just to keep him comfortable. He slept for four days and four nights. When he woke up he asked for tea and complained of feeling hungry. They gave him left over soup and tea. “Well, I am leaving soon,” he announced. He travelled there, he told her. He saw it. “It is of the most beautiful place anyone could ever imagine,” he said, “they sing so well/the melody on the other side.” There let it be he ended, not yet he announced but it’s going to happen anytime I will leave, he was gone in a month’s time. Grandfather was different (before I understood this), he said he was leaving soon. I asked, “Where he was going?” “Where you have ever after happiness, where the dead go,” he replied. “How do you know?” he was asked. My father came to me he said, “At first he was far away and now he is closer he is coming to get me anytime. He came to him to prepare himself, he told me to prepare ahead of time. To set his clothes he’s to wear and moccasins not shoes.”* (Translated from Anishinaabemwin—ALEG Member) *The other thing is … their diet is not really any greens.* (Cochrane Focus Group)
Addressing Systemic Barriers to Culturally Safe Care	*Language and Cultural Inclusion*	*I remember when my grandmother was in the hospital, she wanted cranberries, and not cranberries from the store that you buy, not those ones. Cranberries that you got off out in the bay … They said no. But I still fed her.* (Cochrane Focus Group) *Universities and colleges now have a residential Elder. Why can’t nursing homes have a residential Elder?* (Sudbury Focus Group)

#### Building therapeutic relationships

Building new therapeutic/care provider relationships was described as requiring time, trust, and reciprocity. Elder Otowadjiwan, the ALEG, and the focus group participants all stressed the importance of the approach and taking the time to build a relationship. Important to relationship development is reciprocity. In Anishinaabe culture, sharing information is expected to be bidirectional. Participants encouraged care providers to not only ask questions but also tell something about themselves in exchange. Elder Otowadjiwan pointed out how this is ingrained in the way Anishinaabe people introduce themselves, stating, “I give them my spirit name and my clan, and then I say, they call me by this name every day.” Several participants suggested offering food or a beverage (e.g., a cup of tea, a small breakfast) as important to relationship-building, trust, and starting this interaction in a good way.

All participant groups agreed that building relationships needed to occur over time and would require multiple visits. Care providers need to be attuned to a person’s energy and not push visits or questions when the person being cared for is not ready. In one focus group, it was suggested that if the loved one’s energy feels diminished or hurt, that workers take care of their questions, and offer to return another day. Here, a member of the ALEG shared the word *gmoosha*, meaning “the feeling of knowing that one is in the presence of the loved one’s energy.” The use of the term “loved one” when referring to the person living with dementia is explained below in the Cultural Differences in Word Meaning section.

Building trust includes always being clear about the reason for the visit, and telling the person why the specific types of questions are being asked. Elder Otowadjiwan suggested that workers take a casual and indirect approach, sharing how they came to be in this moment with the loved one. Central to these interactions is active listening, not interrupting them when they are speaking, and paying attention to nonverbal cues such as a head nod or looking up or down.

The importance of humor, kindness, and taking the time needed to listen and speak were emphasized. Advice from Elder Otowadjiwan included, “Slow your speech down; speak as slow as they walk.” It was suggested by many participants that meeting someone new and visiting are considered ceremony in their worldview. A member of the ALEG explained this as *mookseh*, the “beginning of a thought and a powerful spiritual time together.” It was noted that if the Indigenous language is being spoken these are even more significant interactions.

It was repeatedly underscored that it will take time to develop trust, and trust is necessary to explore some of the personal questions in the PIECES of my PERSONHOOD tool. Elder Otowadjiwan stated, “It takes time and courage to tell the complete truth.”

### Value Indigenous Language

Two significant and related subthemes emerged from the various participant groups related to valuing Indigenous language: Indigenous language use and cultural differences in word meaning.

#### Indigenous language use

Facilitating the ability of the person being cared for to use their first language was considered vital to both the comfort of the older adult and to the accuracy of the information being relayed. That is, the truth can be more easily conveyed in their first language. Participants agreed that many older Anishinaabe in northern Ontario are first-language speakers, with English or French being second languages. It was considered important that interpreters be available when asking these biographical questions. The team was also cautioned that there are variations in the specific languages (primarily Cree and Ojibwe) due to regional dialects and first speakers who speak “the old language.” It was thought to be important for care providers to know that those speaking the old language may not be understood well by those who have learned the language more recently.

#### Cultural differences in word meaning

A second related topic that emerged in all informant groups was that of discrepancies between Indigenous and mainstream definitions and terminology that related to aging and caregiving. Elder Otowadjiwan shared teachings on love that indicated Indigenous people being assessed should be referred to as “loved ones,” a term that reminds those providing care that this person is loved by someone. However, in mainstream practices, care providers are guided away from the term “loved one” to avoid assumptions about that person’s relationship to the person being assessed (Alzheimer Society of Canada, 2017). In another example, the terms “care partner” or “family care partner” are increasingly encouraged for use; however, the informants we spoke with strongly endorsed “caregiver” because to offer care to another is considered a great gift in Indigenous culture.

The ALEG cautioned that how people refer to their spouse may seem insulting or unusual to non-Indigenous providers, but that is not necessarily the case (Table 2). For example, “old man” or “old lady” when translated are respectful terms. Independence also took on a nuanced meaning. We learned that older Indigenous adults value doing as much as they can themselves for as long as they can, yet still invite others to assist with certain tasks to support their independence. This means that older adults may direct a few different “helpers” to support them in completing tasks that are becoming difficult, and yet retain independence because they have complete control of their circle of care. Participants shared that an Anishinaabemwin speaker would never ask, “Who cares for you?” Rather, they would acknowledge that the loved one does as much as they can and ask, “Who picks up the little pieces of things that you cannot do anymore?” translated literally to, “Who catches that for you?”

Finally, the term “memory loss” as it is used in relation to a dementia/neurocognitive disorder diagnosis in Western medicine was not aligned with Indigenous beliefs. A community-based researcher and author (KP) shared the term *ngwosh kaa ni we ga kendung*, which means that “memories are covered or buried, not lost.” This was described as a way of viewing the loved one as a whole—all their knowledge and personality remained intact, but aspects were buried, indicating a need for kindness, love, and understanding. This understanding was presented and validated in all three focus groups.

### Understand Indigenous Trauma

Reviewing the personal history questions in the PIECES of my PERSONHOOD tool led to discussions from all informant groups about the painful colonial history suffered by Indigenous peoples in Canada and elsewhere. Participants shared that many of the older adults who would be asked the questions in this assessment had either directly or indirectly been impacted by residential schools, the Sixties Scoop, and other policies in the Indian Act that led to displacement from their community, the land, traditional food sources, and Indigenous healing practices. Further, it was shared that the older adults and their caregivers continue to be exposed to policies that separate children from their families and force community members off-reserve for employment. Consequently, it was felt that many of the life history questions that ask about where people had grown up, their employment history, and family members may evoke an unintended trauma response. Also captured in this category is deep mistrust of institutions and White care providers. Two key subthemes emerged: triggers and approach.

#### Triggers

Participants shared that Indigenous people from their communities may have a fear of long-term care homes or other congregate environments. Some participants were survivors of the residential schools or the foster care system and shared that they had either been taken away from their homes and families or had watched loved ones disappear. Focus group participants connected this experience with their present-day concern they would be taken away from their communities and placed in long-term care without their consent (Table 2).

Participants in the focus groups described how White service providers operated the residential schools and carried out the policies of the Sixties Scoop. Though it is not their intention, White service providers represent this legacy in their current work. An example was shared in Serpent River, in which a participant explained that she still feels fear when seeing White service providers with black notebooks because she associates them with traumatic experiences interacting with child welfare services many years ago.

In another example, the ALEG shared that asking about a person’s job or employment history may also trigger sadness. They expressed that there is a differing interpretation of “work” in Anishinaabe culture compared to Eurocentric cultures. The focus group in Cochrane also confirmed that this is consistent in the Cree culture. Paid work did not translate as a source of joy or meaning in Anishinaabe or Cree culture but was viewed as a painful burden that caused disconnection because many older Indigenous adults had to leave their communities and families to find work elsewhere due to lack of employment opportunities and housing on-reserve. An ALEG member stated that asking a loved one about their past work life translated to, “What exactly was the activity you did while you were away working on that job, removed from and no longer in a relationship with your family or community?” However, some focus group members also spoke fondly of more modern work arrangements and explained that they would want to have the opportunity to discuss these situations.

Most participants agreed that asking about family, including spouses, is a final example of a potentially triggering question. They felt that, depending on the type of relationship the loved one had with their spouse, asking about them could be inappropriate. In two focus groups (North Shore and Cochrane), participants talked about arranged marriages and different definitions of marriage, and in all three areas, participants indicated that workers should not ask directly about spouses or partners.

#### Approach

During difficult discussions, participants thought that questions should be revised so that direct questions concerning trauma were avoided and that care providers should be trained to “let them lead” when these topics arise. It was also suggested that some of the questions may be more appropriate to ask the caregiver rather than the person being cared for.

Participants also emphasized the importance of training the care providers on the context and deeper meaning behind the term “trauma” in Indigenous populations. In Anishinaabemwin, the way that trauma is described does not allow the term to be decontextualized or stripped of deeper meaning. As stated by an ALEG member, “Someone who has trauma almost experiences a tear up of every essence of who they were, so much that the pieces could not be put back together to be any kind of whole ever again.” This practice of respecting a person’s trauma story without asking that they relive it was preferred in all focus groups and illustrates the importance for care providers to know those potential triggers and incorporate key approaches into care planning.

### Respect Cultural Values and Understandings

Cultural understandings and shared colonial history are interwoven into all of the thematic categories that have emerged as central to creating a culturally safe adaptation of the PIECES of my PERSONHOOD tool. However, Indigenous culture in itself also emerged out of these discussions as a key consideration.

#### Wholism

The findings from the sessions highlighted how the Indigenous conceptualization of “wholism” could transform the structure of the tool into something more familiar to Indigenous people and a tool that would provide more information important to their care. We adopt the spelling of wholism as opposed to holism to emphasize and honor the Indigenous conceptualization of the *whole*. In assessing the overall structure of the PIECES of my PERSONHOOD tool, all participant groups felt that the tool could benefit from the inclusion of full sentences that offered specific context and improved flow instead of direct prompts. In addition, they felt that there were too many questions. For example, PIECES of my PERSONHOOD begins with prompts for *name* and *preferred name*, *family background*, *pets and animals*, and *significant persons in life and relationships.* However, this was not congruent with the way participants would normally introduce themselves. In their cultures, this information is combined in an introduction to oneself that includes sharing your name, spirit name, community, clan, relatives, and other details to orient themselves to the rest of the group. They suggested instead that these questions be combined into one introductory section titled “Who I Am.” Participants also noted that sharing of spirit names, which often happens when introducing oneself, can be deeply personal. As stated during a focus group, “Our names are very sacred. So if you’re going to ask that question, make sure you’re going to be really attentive to what they say and everything, because that’s really important” (North Shore focus group). Likewise, it was thought that the multiple questions around preferences in the tool (*mealtime preferences*, *sleeping and waking preferences*, and *socialization preferences*) could all be answered better by asking about “A Day in My Life.” They felt this would allow for a fuller description of all the person’s needs and preferences rather than predefined categories related to only food and sleep.

#### Cultural and spiritual knowledge

Although the participants wished for a more expansive view on preferences than just food and sleep, they also shared specific cultural knowledge related to these topics. Participants suggested paying special attention to food preferences and if possible, discussing land-based food preferences and accommodating those when possible. They felt that discussion of land-based food could also elicit preferences around texture and color of food that may not come forward otherwise. It was noted that especially for the Cree, the traditional diet was largely meat based, which would factor into their likes and dislikes. In relation to sleep, participants wanted care providers to know that they should not directly ask about dreams, as dreams are considered personal and spiritual experiences. Importantly, participants placed a high value on their ability to continue wellness practices, such as smudging and using specific plant-based medicines, while in any formal care setting such as hospitals or long-term care.

Our conversations about spirituality and coping sparked discussions about end-of-life care in all the focus groups. Participants shared the concept of “ever after happiness” as vital to their final transition in life. As part of this transition from life here to life in the spirit world, it would be expected that the person being cared for would see and play with relatives and ancestors who had already passed. They may also begin preparing their belongings for the journey. This would be comforting to the individual and needs to be respected as part of their care model. However, these behaviors are often misunderstood by care providers as confusion, hallucinations, or delusions. The importance of ensuring providers could distinguish between these visions and journeys and clinical delirium was also emphasized.

#### Cultural values

Several recommendations were made by participants to embed Anishinaabe values into the questions and process—for example, as already discussed, the values of *reciprocity* and *honesty/truth* in relationship development and *love* in the act of caregiving. Another Indigenous value that was important to participants was *humility*. Elder Otowadjiwan shared, “We’re not used to talking about ourselves, we’re humble people, it’s about belonging to a community, relationships are important.” The ALEG stated that, “We are humble people, not boastful; belonging is valued.” In response, some participants from different groups expressed concerns about the inclusion of pride-related questions from the PIECES of my PERSONHOOD tool. They believed that these prompts should be removed because they contradict the cultural value of humility by encouraging the discussion of accomplishments, honors, and feelings of pride.

### Address Systemic Barriers to Culturally Safe Care

Focus group participants discussed changes that could be implemented to improve the care of older Indigenous adults that moved beyond modifying the PIECES of my PERSONHOOD tool. Many of these are aligned with previous observations on the tool itself but are presented as a separate theme because they spoke to systemic (policy and procedural) changes needed to address overall biases and barriers to culturally appropriate care.

#### Language and cultural inclusion

Translation services resurfaced as a top priority, not specific to the use of the tool but more generally to accommodate older Indigenous adults in all aspects of their care. Building on previous discussions about understanding cultural influences on preferences and communication styles, participants encouraged NEBSO to advocate for further inclusion of Indigenous culture in long-term care homes in the northeast region of Ontario. Specific suggestions from participants included offering and allowing wild food, conducting activities older Indigenous adults may be familiar with, and creating talking circles. It was deeply important to participants that smudging, sacred medicines, and other spiritual items and practices be made available and visible in long-term care homes. The Sudbury focus group stated, “They don’t even have a room for that smudging. We don’t have a room for the Natives or anything in there. It would be nice if they had that.” Participants stressed that having these practices be open and visible is important because these practices were historically banned by the government, and older Indigenous adults may not be sure if they are allowed to engage in or request these ceremonies. It was suggested that integration of ceremony into these settings could be accomplished by partnering with nearby Indigenous organizations, having a designated knowledge holder/Elder in residence, and incorporating activities and outings that engage older Indigenous adults in long-term care with the natural world such as nature walks and medicine walks. For example, the Sudbury focus group described being invited to be spiritual advisors: “When I used to work here [the Friendship Centre], some nursing homes would call about every three months and we would go in as spiritual advisors.”

#### Training

Participants felt that training on the revised PIECES biographical tool would be important to making the tool appropriate and safe for use, but they went further in suggesting that the long-term care staff be provided with regular training concerning the provision of culturally safe care and trauma-informed approaches in an Indigenous context with an emphasis on colonial history.

## Discussion and Implications

Over 36 months, the research team and community partners iteratively examined the themes from data collection and used consensus to continuously build findings into revised versions of the PIECES of my PERSONHOOD tool with the aim of creating a culturally safe biographical tool for Indigenous populations in northeastern Ontario. This resulted in the new PIECES of my RELATIONSHIPS biographical tool. Using Indigenous methodologies and a cultural safety framework, the tool was transformed from 21 individual questions into five domains: *Who I Am*, *A Day in My Life, What Keeps Me Going, How to Keep Me Safe,* and *How to Care for My Whole Being* (Table 1). The original tool was expanded with three additional resources to support a culturally safe approach to administering the tool, including (1) a supplemental caregiver/family tool, (2) an administration guidebook that includes cultural safety and preferred communication style guidelines, as well as (3) a quick guide to approach.

### Personhood to Relationships

The adapted tool was renamed PIECES of my RELATIONSHIPS to highlight the importance of community and relationships among potential Indigenous care recipients, underscoring the significance of the relational worldview held by the Indigenous people who would be receiving care. In refocusing the tool from *personhood* to *relationships*, our adaptation reflects prior work that highlights the importance of understanding personhood in Indigenous context as relational and collective (Kirmayer et al., 2003; Waid & Frazier, 2003; Webkamigad, Warry, et al., 2020).

### Trauma

A significant finding related to cultural safety was the importance of using trauma-informed approaches when working with loved ones. The research team sought to re-contextualize the term “trauma” by placing the current experiences of older Indigenous adults in northeastern Ontario within the larger fabric of the colonial story in this area. The training guide contains sections detailing the impacts of colonialism and the importance of trauma-informed approaches. In this guide, colonialism is presented as contemporary and ongoing, and is followed by a discussion of trauma-informed approached with Indigenous populations. Trauma-informed approaches are emerging as best practice in mental health and social services in Canada (Smith & Abel, 2016).

The revised tool also altered the original tool’s *significant low point(s) in life* domain to a more trauma-informed approach and specific wording in the new tool was carefully considered. For example, questions that seemed benign to a Western audience, such as *where were you raised*, were identified as being potentially traumatic considering the history of residential schools.

### Cultural Safety

In recent years cultural safety has gained momentum as a critical shift in our approach to cross-cultural care for Indigenous people. This research sought out to address culturally safe care for older Indigenous adults by engaging with Indigenous community and organizational partners to inform, direct, and confirm the adaptation and creation of new clinical tools to be used in an institutional setting with older Indigenous adults. In a review of cultural safety in health and dementia care by Chakanyuka and colleagues (2022), “power and power relations” are highlighted as integral components of culturally safe care. In our work, the research process itself represented the first step in addressing the historical power imbalance between NEBSO and Indigenous communities. Cultural safety requires health care organizations to shift power, operational policies, and procedures to address personal and systemic biases and prejudices that impact the quality of care (Curtis et al., 2019). This includes cultural safety training for staff and service providers to provide education and change habits that lead to the application of culturally safe practices. The revised PIECES of my RELATIONSHIPS toolkit provides a mechanism for this to occur within NEBSO. The supplementary materials provide training and education on the history of relations with Indigenous populations and promote awareness of cultural norms and understandings relevant to their care (Jacklin et al., 2018). It is designed to be culturally congruent with a relational focus and relationship development which challenges Western biomedical norms. It also goes further and embeds the practice of culturally safe care into the organizational culture and structure by supporting the use of the tools.

The recommendations for additional organizational change that came forward during the research ranged from structural changes for long-term care homes to allow for greater presence and incorporation of Indigenous culture, to the desire for NEBSO to become advocates and continue to work closely with Indigenous communities to ensure their voices are heard and needs are met. Transforming these recommendations into actions can only take place if the relationships between NEBSO and the Indigenous communities continue to be prioritized and supported.

#### Limitations

Despite recruitment efforts, 22 focus group participants were women while only 2 were men. This gender imbalance may mean that certain male-socialized behaviors, traditions, and/or ideas are not fully represented.

## Conclusion

The adaptation of the original practice tool resulted in a set of culturally safe and trauma-informed practice tools to use with Indigenous adults and their families at a regional level. The CBPR and Indigenous methodologies allowed the team to create an adapted tool for the diverse Indigenous populations represented in northeastern Ontario. The name of the tool was changed to PIECES of my RELATIONSHIPS to emphasize the importance of a relational approach to care and the need to understand the person in the context of their relationships. The approach to the project and the partnership with NEBSO allowed cultural safety to be addressed at multiple levels including the changes to the practice tools for cultural alignment, training of staff through the supplementary materials, structural changes supporting the implementation of the materials, and an ongoing commitment from the NEBSO team to continue to foster a dialogue and relationship with Indigenous communities in the region. The findings demonstrate inherent incongruencies between underlying Western perspectives concerning patient care and what is required to provide culturally safe care to Indigenous residents. These findings support the need to carefully evaluate the cultural safety of other assessments and practices for older Indigenous adults in other health care settings.

## Supplementary Material

gnad176_suppl_Supplementary_Tables_S1

## Data Availability

Our Institutional Research Board approvals do not include the ability to share raw or aggregate data including analytic materials in order to protect Indigenous data sovereignty. Some materials may be made available to other researchers by contacting the corresponding author who can facilitate discussions with the Indigenous communities involved in the study to inquire about specific requests. Codes and coding schemes are available in the supplementary materials. This research was not pre-registered with any registry.
