# Supplementary material for: PIECES of My RELATIONSHIPS: The Cultural Adaptation of a Biographical Assessment Tool for Indigenous Older Adults in Canada
Source: Gerontologist. 2023 Dec 27;64(7):gnad176. doi: 10.1093/geront/gnad176 (PMC11194628; doi:10.1093/geront/gnad176)
Supplement: gnad176_suppl_Supplementary_Tables_S1 [file gnad176_suppl_supplementary_tables_s1.docx]

**Online Supplementary Material**

**Supplementary Table 1:** Emergent themes

| **Main Theme** | **Subtheme** | **Topics** |
| --- | --- | --- |
| Practice a relational approach to care | Relationships with others | - Wholism - Relationships with animals |
|  | Building therapeutic relationships | - Cultural values informing relationship development - Reciprocity - Energy - Non-verbal cues - Ceremony - Trust |
| Value Indigenous languages | Indigenous language use | - First language speakers - Translators - The ‘old language’ - Truth telling |
|  | Cultural differences in word meaning | - Loved ones - Caregivers - Old lady/man - Independence - Memory loss |
| Understand Indigenous trauma | Relationship between triggers and institutional care | - Historical trauma and loss - Residential schools - Sixties Scoop - White people - Indian Act policies - Employment |
|  | Approach | - Indigenous trauma - ‘Let them lead’ |
| Respect cultural values and understandings | Wholism | - Consolidation of questions |
|  | Cultural and Spiritual Knowledge | - Preferences - End of life |
|  | Cultural values | - Reciprocity - Honesty/Truth - Love - Humility |
| Address systemic barriers to cultural safe care | Language | - Translators/interpreters |
|  | Culture | - Incorporation of practices - Make it visible |
|  | Training | - The PIECES tool - Colonialism |
